# Community knowledge, attitudes and practices towards malaria in Ha-Lambani, Limpopo Province, South Africa: a cross-sectional household survey

**DOI:** 10.1186/s12936-021-03724-z

**Published:** 2021-04-17

**Authors:** Mukhethwa Munzhedzi, Elizabeth T. Rogawski McQuade, Jennifer L. Guler, Piper E. Shifflett, Sara Krivacsy, Rebecca Dillingham, Pascal O. Bessong

**Affiliations:** 1 HIV/AIDS & Global Health Research Programme, and Department of Microbiology, University of Venda, Limpopo Province, Thohoyandou, South Africa; 2 Department of Public Health Sciences, University of Virginia, Charlottesville, VA USA; 3 Department of Biology, University of Virginia, Charlottesville, VA USA; 4 Center for Global Health Equity, Department of Infectious Diseases and International Health, University of Virginia, Charlottesville, VA USA

**Keywords:** Malaria, Knowledge, Attitudes and practices, Ha-Lambani, Limpopo Province, South Africa

## Abstract

**Background:**

Malaria remains a global health concern and is endemic in Limpopo, Mpumalanga and KwaZulu Natal Provinces of South Africa, which aims to eliminate malaria by 2025. Community engagement plays a significant role in improving the acceptability and effectiveness of programmes aimed at reducing malaria transmission. The success of such intervention efforts depends on the knowledge, attitudes and practices (KAP) of the community, and understanding the KAP of community residents may support malaria control efforts in the locality. In this context, a cross-sectional household survey to assess community KAP on malaria transmission and prevention in the Ha-Lambani village, Vhembe District, Limpopo Province was conducted.

**Methods:**

Data were collected between November 2018 and May 2019 by questionnaire of 261 consenting adults (213 females and 48 males, aged between 18 and 95 years) selected from different households. Also, a focus group discussion among 13 randomly selected participants was conducted. Pearson’s Chi Square test was used to determine statistical differences by village.

**Results:**

Study participants (100%, 261/261) were aware of the presence of malaria in their community and 95% associated it with mosquito bites. The local health clinic was the most prominent source of malaria information (85%). Only 22% correctly identified headache, chills and fever as the three most common symptoms of malaria. The majority of participants (98%) knew that effective medication for malaria is available and had a positive treatment-seeking behaviour. Knowledge of malaria prevention measures was high (82%); contrarily, 97% of respondents did not sleep under a bed net the previous night. The focus group data concurred with these results and also revealed that poor bed net use resulted from lack of access to bed nets because community residents could not afford them.

**Conclusions:**

The study demonstrates that participants have appropriate knowledge about malaria transmission and a positive treatment-seeking behaviour. However, economic barriers are responsible for the inadequate use of bed nets. Therefore, distribution of bed nets to the community should be considered to improve practice of malaria prevention measures. Furthermore, knowledge of signs and symptoms and appropriate malaria treatment was limited, and initiatives to improve awareness on these topics should be continued.

**Supplementary Information:**

The online version contains supplementary material available at 10.1186/s12936-021-03724-z.

## Background

Malaria incidence in South Africa has been estimated at 20 cases per 100,000 population in 2015. In 2017, there were 22 517 cases and 301 deaths. Of these cases, 8700 were from imported malaria due to cross border movements. However, in 2018, the numbers have been reduced to 9540 cases, 5742 imported cases and 69 deaths [1]. Amongst the known species, *Plasmodium falciparum* is the predominant species associated with severe and fatal disease and remains the causative agent of up to 90% of malaria cases in South Africa [2, 3]. Malaria is restricted to the low altitude areas of three provinces, namely Limpopo, Mpumalanga and KwaZulu-Natal, where it is endemic. Limpopo shares borders with Zimbabwe and Mozambique; Mpumalanga shares borders with Eswatini and Mozambique, while KwaZulu Natal shares boarders with Eswatini and Mozambique, respectively. These provinces experience seasonal transmission of malaria [2, 4–7]. The entire Limpopo Province was historically at risk for malaria; however, through vector control by indoor residual spraying (IRS) introduced in the 1940s, malaria is now restricted to the eastern and northern areas of Mopani and Vhembe districts of the Province [2, 8]. Thus, due to conducive environmental conditions as well as cross border movements, only certain provinces of South Africa experience seasonal malaria transmission occurring during the summer months, September to May.

Between 2015 and 2018, a dramatic increase in malaria cases was observed in Limpopo province, with a more than tenfold increase across the summer transmission seasons (September-May). In March 2017, the Ministry of Health reported a total of 4092 cases of malaria for the Vhembe and Mopani districts with 33 deaths. Furthermore, malaria cases were also reported in Provinces that are not endemic for malaria like Gauteng (Doornpoort) and Northwest (Swartruggens) [9]. Case numbers collected from the primary health clinic in Ha-Lambani in Limpopo Province increased from 6 to 190 cases during the malaria seasons of 2015–2018. No studies on malaria knowledge, attitudes and practices (KAP) have been done in this area.

Community knowledge is assessed in order to determine the extent of overlap with biomedical concepts. It plays a significant role in improving the acceptability and effectiveness of programmes aimed at reducing malaria transmission. Understanding KAP of residents of a community regarding malaria can assist in the reformulation of control strategies and form the basis of appropriate health education messages as well as obtain and maintain the community’s participation in surveillance and control activities [10–14]. Failure to consider such beliefs and perceptions of the planned programmes may result in negative attitudes or practices and eventually result in failure to achieve the intended goal [14, 15].

In the context of sporadic high transmission of malaria with recent increases in burden in Ha-Lambani, it is important to understand the KAP of the residents of the community to gather data that may support local malaria control efforts. In this context, a cross-sectional household survey to assess community KAP surrounding malaria transmission and prevention in Ha-Lambani, Vhembe District, Limpopo Province was conducted.

## Methods

### Study design and setting

**Fig. 1:**
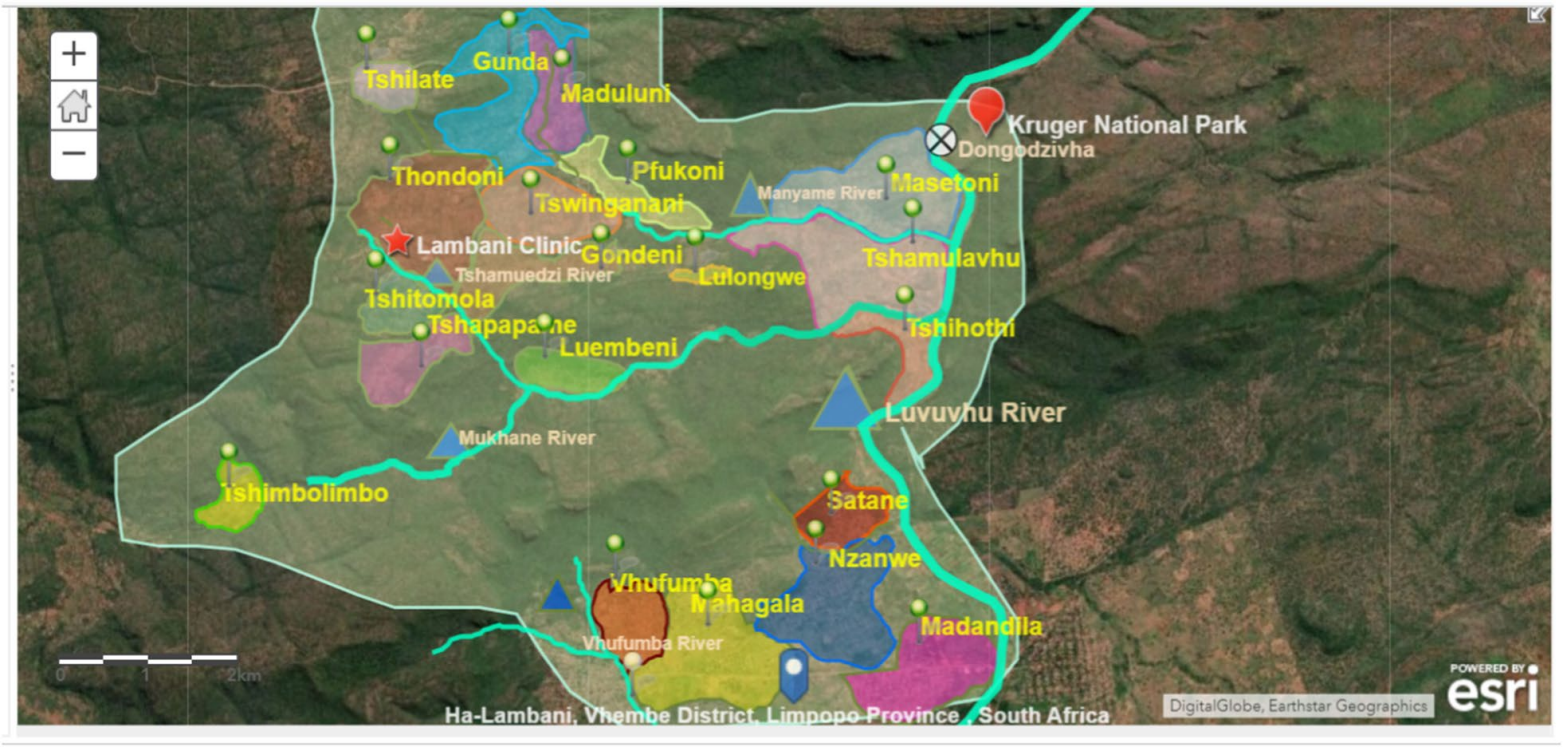
Map showing the villages that make up Ha-Lambani, South Africa. The villages are represented by the green pushpins. The villages with the highest number of cases of Malaria, Tshihothi and Masetoni, lie near Luvuvhu River. Blue triangles represent rivers, red star represents the local clinic, and the red point the Kruger

A descriptive, cross-sectional household survey was conducted in Ha-Lambani community in the Vhembe district from November 2018 to May 2019. Ha-Lambani (22.7108° S, 30.8442° E), is a malaria-endemic area, at a low altitude of 605 m above sea level. Figure 1 shows the spatial map of Ha-Lambani area.

Briefly, Ha-Lambani area is made up of 20 villages. It is a community of low-socioeconomic status, with a population of 15,078 people in 3015 households. The majority of residents are subsistence farmers, casual workers and pensioners. It is approximately 16 km away from the Kruger National Park (one of Africa’s largest game reserves), an area considered low-to-medium risk for malaria transmission during summer months. Also, it is approximately 98 km away from Pafuri Rest Camp in Mozambique, and 176 km from the Zimbabwean Beit Bridge border post. The study area used for the KAP assessment comprised three villages, Masetoni, Tshihothi and Tshamulavhu, which had recently experienced significant increases in malaria cases based on statistics from the local health centre.

### Sampling strategy

In the current study, 261 participants consented to participate and each was administered a free malaria rapid diagnostic test (RDT). Consent was sought from each available household head then from any adult available and willing to participate. The three villages had 584 households with a total population of 2995 in 2018. Overall, about 90% of the available households were sampled. For each household, only one consenting adult (≥ 18 years old) was selected for interview.

### Data collection

Two female homebased care workers from the local clinic were recruited and trained to support the recruitment of study participants and conduct the interviews. Trained field workers administered a questionnaire that was both in English as well as in Tshivenda (the local spoken language) to respondents that were 18 years and older. The survey questionnaire was open ended and had these sections: demographics of respondent; treatment-seeking behaviour; and knowledge, attitude and practices related to malaria transmission and disease. The survey questionnaire was piloted before use to ensure comprehensibility, clarity and appropriateness. After each interview, an informational pamphlet on causes of malaria, transmission, prevention and treatment, written either in English or Tshivenda, was given to the household. This pamphlet was developed by collating information on malaria transmission from Tshaulu Malaria office, Thohoyandou Health Centre and Malaria Control Programme office in Thohoyandou, Limpopo Province. The pamphlet was approved by the Limpopo Provincial Department of Health. Pamphlets were also provided to outpatients of the local clinic and to the community at the end of the study, to create awareness in the community at large.

A questionnaire and a focus group discussion (FGD) were chosen as the data collection methods to ensure comprehensive methods of data collection. The questions on the interview guides were checked by other investigators not associated with the current study, who have relevant knowledge and expertise to ensure that questions were correctly phrased and that they captured all the necessary information needed for the study.

### Focus group discussion

After individual interviews were performed, a respondent moderator focus group discussion was conducted once wherein the researcher recruited a participant in the group to take the role of a moderator. The purpose of the focus group discussion was to obtain more information in order to confirm what was already observed from the individual questionnaires as well as to see if other themes or ideas emerged. This discussion comprised 13 randomly selected individuals: 12 individuals from the study site who were already participants (one of which served as the moderator) and 1 person who was an investigator of the current study (who served as an assistant moderator). The role of the moderator was to facilitate the discussion, ensure smooth flow of the discussion and participation by all; as well as ensuring that participants did not deviate from the topic under discussion [16]. The assistant moderator served as a scribe to record the participants’ responses once there was a consensus as well as to tape record the participants responses in order to confirm or compare with what was written.

### Quality assurance and data analysis

Quality of the data was assured by double checking the entries on Microsoft Excel version 2010. Statistical analysis was done using social science statistics. Qualitative data was analysed using descriptive statistics. Frequency distributions and percentages of variables were used to describe the sample population, quantify knowledge of malaria, attitudes and practices associated with malaria transmission and prevention. Narratives from FGD qualitative data were coded and managed using themes that address key issues based on study objectives. Coding and coding consistency checking was carried out. A list of codes was developed, reviewed and grouped into themes and categories for analysis [17]. The following main themes formed the focus of the findings: demographics of respondent; malaria knowledge (symptoms, treatment, prevention), attitude and practices; treatment-seeking behaviour and awareness related to malaria transmission and disease. Pearson’s Chi square test was used to determine the statistical significance of difference of relative frequencies between villages, and differences by gender, age, educational level and total household income. Values were considered statistically significant when *p* < 0.05.

### Operational definition of selected variables

- Married: One was considered married either lawfully, traditionally or by living with husband or wife.
- Home-based care workers: Those who provide routine personal care and assistance with activities of daily living to persons who are in need of such care due to effects of ageing, illness, injury, other physical or mental condition in private homes and other independent residential settings. They are usually members of the local community, give health talks to their patients and their families as well as cooperate with the advice and support from the trained health workers from the local health clinic.
- Field worker: Someone who is trained to work outside of the office and travel to different locations to recruit study participants (administering questionnaires and conducting interviews for data collection).
- Musuzungwane: Scientifically known as *Lippia javanica*, is a plant that is traditionally used to ward off mosquitoes.
- Bunganyunyu plant: A plant that is traditionally used to ward off mosquitoes.
- Mudinyane: Another alternative name for malaria that is used by some of the current study participants.

### Ethical consideration

The study protocol was approved by the Research Ethics and Clinical Trial Committee of the University of Venda (protocol number: SMNS/18/MBY/09/0507). Permission to use local health facility was obtained from the Limpopo Provincial Department of Health (Ref: LP_2018 08-020). Also, permission to conduct the study in the community was obtained from the community leader of Ha-Lambani area. Informed consent was obtained from each study participant before proceeding with the survey. Anonymity of study participants was adhered to.

## Results

### Demographic characteristics of the study population

A total of 261 questionnaires were completed, with no refusals, and 100% of the questions were answered. Females accounted for approximately 82% (213/261) of the total study population. The ages of the participants ranged from 18 to 95 years, with a mean, median and standard deviation of 44.1, 40 and ± 18.1 respectively. Most of the study participants were mothers (70.1%, 183/261). The majority of respondents, i.e. 71.6% (187/261), were unemployed (casual workers and pensioners) and more than three quarters of them, i.e. 79.3% (207/261), had at least primary school level education. In addition, 1.9% (5/261) of the participants reported that they had malaria (tested by Rapid Diagnostic tests) within the previous 3 months. One focus group discussion was conducted after the individual interviews and constituted of men and women aged between 21 and 79 years old. Additional file 1: Table S1 represents the demographics of the study participants.

## Malaria knowledge

### Participants’ knowledge on the transmission of malaria

Of the 261 participants, all (100%; 261) had heard about malaria, with about 95% (247/261) correctly associating malaria with mosquito bites. However, a small number of the participants had no knowledge about malaria transmission at all and this proportion differed significantly across the villages: Masetoni (5.8%), Tshamulavhu (1.5%), Tshihothi (12.2%) (P = 0.01). When asked about their general view of malaria, the majority of study participants (93.1%; 243/261) who had heard about malaria further demonstrated appropriate knowledge of malaria by recognizing it as a problem, stating that it is dangerous, that it can kill if not treated early, it’s a terrible or frightening illness; or by directly stating that it is a problem. However, only 1.9% (5/261) and 3.1% (8/261) of the study participants mentioned malaria signs and symptoms, and prevention methods, respectively. Although majority of study participants recognize malaria as a health problem in the community, only 2% (5/261) recognized the proximity of Kruger National Park to the study area as an important risk factor for the transmission of malaria. Of note, 97% (253/261) of participants who believe that malaria is dangerous and kills or see it as a health problem have been resident in the study area for at least 5 years. Additional file 2: Table 2 shows the details on the knowledge and perception on malaria transmission of study participants.

The study participants gave a wide range of sources of malaria information (Table 1). However, clinics (health centres) (84.7%; 221/261) were the most prominent sources. Family was also an information source in some villages, but not others (P = 0.03), while other sources were not commonly reported in any of the villages (indoor residual spraying (IRS) team, television, home-based care workers, neighbours or newspapers, P = 0.85). Additionally, friends were the least common source of malaria information (1.1%; 3/261).

**Table 1:** Reported sources of malaria information by village

Village name	Masetoni n = 86	Tshihothi n = 41	Tshamulavhu n = 134	Total n = 261	p value
Source of malaria knowledge	n (%)	n (%)	n (%)	n (%)	α = 0.05
Radio	0(0)	3(7.3)	3(2.2)	6(2.3)	–
School	3(3.5)	0(0)	3(2.2)	6(2.30	–
Clinic	78(90.7)	32(78.0)	111(82.8)	221(84.7)	P = 0.13
Friends	1(1.2)	0(0)	2(1.5)	3(1.10	–
Family	1(1.2)	5(12.2)	12(9)	18(6.9)	**P = 0.03**
Other sources^e^	3(3.5)	1(2.4)	3(2.20	7(2.7)	P = 0.85
Total	86(100)	41(100)	134(100)	261(100)	–

Other sources^e^ of malaria information included Indoor residual Spraying team, television, home-based care workers, neighbors or newspaper. Chi-square test for differences in prevalence across villages

### Participants’ knowledge on malaria symptoms

**Fig. 2:**
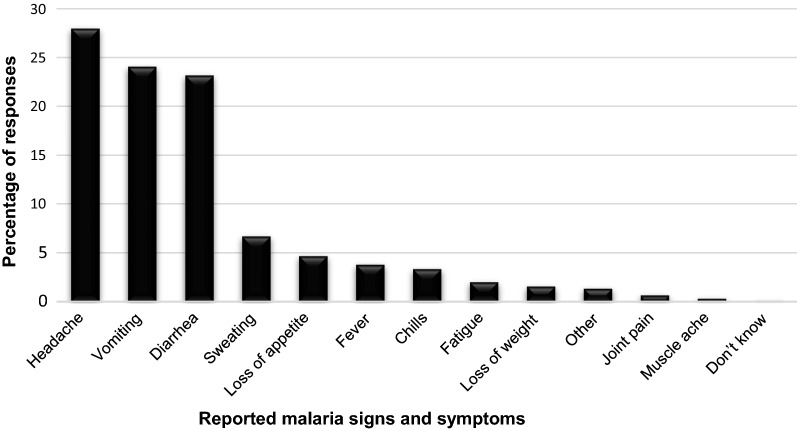
Respondents’ knowledge of malaria signs and symptoms (other included dizziness, stomach pains, loss of weight, sore eyes and being delusional)

Malaria signs and symptoms such as headache, vomiting and diarrhoea were the most frequently reported. However, participants also mentioned sweating, loss of appetite, fever and chills; and to a lesser extent fatigue, loss of weight, joint pains and others. One participant (0.4%, 1/261) did not know of any symptoms of malaria (Fig. 2). Overall, about 99.2% (259/261) correctly identified at least one of the three most common symptoms of malaria (headache, chills and fever [1]). However, only 21% (56/261) of the participants were able to correctly identify all 3 of these most common symptoms of malaria.

### Participants’ knowledge on malaria treatment

The participants’ knowledge of malaria treatment was low, with only one participant mentioning quinine as a drug to treat malaria. However, a majority of the participants knew that effective treatments for malaria are available (98.08%; 256/261) and 83.5% (218/261) mentioned tablets/pills as medication for malaria treatment. Only 1.5% (4/261) of the respondents had no knowledge of any form of malaria treatment (Table 2).

**Table 2:** Reported knowledge of malaria treatment of participants by village

Village name	Masetoni n = 86	Tshihothi n = 41	Tshamulavhu n = 134	Total n = 261
What do you know about malaria treatment?	n (%)	n (%)	n (%)	n (%)
Pills	77(89.5)	31(75.6)	110(88.8)	218(83.5.)
Quinine	0(0)	0(0)	1(0.74)	1(0.74)
Clinic	7(8.1)	3(7.3)	4(3)	14(5.4)
Drip (intravenous)	0(0)	1(2.4)	1(0.7)	2(0.8)
Pills and/ medicine	1(1.2)	2(4.9)	3(2.2)	6(2.3)
pills, drips(intravenous)	1(1.2)	2(4.9)	5(3.7)	8(3.1)
Hospital	0(0)	1(2.4)	0(0)	1(0.4)
Other^f^	6(7)	0(0)	1(0.7)	7(2.7)
None	2(2.3)	1(2.4)	1(0.7)	4(1.5)
Total	86(100)	41(100)	134(100)	261(100)

Other^f^ reported treatment options included mentioning that it works, those with malaria should adhere to it and that it is helpful

## Attitudes and practices towards malaria

### Treatment-seeking behaviour of participants

A total of 97.7% (255/261) of the participants had a positive attitude regarding seeking treatment by stating that they would take their child to clinic if they had symptoms such as fever. However, a small proportion (1.5%; 3/261) reported that they would either use Panado (paracetamol), pray or talk to their pastor (Table 3).

**Table 3:** Reported treatment-seeking behaviour of participants by Village

Treatment-seeking behaviour (when seeking child care e.g. when a child has fever)	Masetoni n = 86	Tshihothi n = 41	Tshamulavhu n = 134	Total n = 261
	n (%)	n (%)	n (%)	n (%)
Consult at the clinic	84(97.7)	41(100)	130(97)	255(97.7)
Give him Panado	0(0)	0(0)	1(0.7)	1(0.4)
Praying, talk to the pastor then clinic	0(0)	0(0)	2(1.5)	2(0.8)
Others^g^	2(2.3)	0(0)	1(0.7)	3(1.1)

Other^g^ steps to take when seeking childcare included calling the ambulance, giving the child boiled lemon leaves to drink and breastfeeding the child more

### Participants’ knowledge and practices on malaria prevention

Most of the study participants were aware of malaria prevention measures (81.9%; 214/261). However, the rest of the study participants either did not know or mentioned an incorrect malaria prevention measure(s) such as using clean water, eating clean food and drinking soft drink. Amongst other prevention measures, wearing long-sleeved clothes (39.1%, 102/261) proved to be the most prominent in the study population; this was followed by using bed nets (23.8%, 62/261) and these proportions differed significantly across villages: Masetoni (48.8%), Tshamulavhu (44%), Tshihothi (2.4%; p = 0.00); Masetoni (27.9%), Tshamulavhu (26.1%), and Tshihothi (7.3%), respectively (p = 0.03).

Also, most (85.4%; 223/261) of the study participants correctly reported removal of stagnant water and removal of used cow dung (for decorations), proper disposal of empty cans or keeping their compound clean as ways to prevent mosquito breeding. Many of these proportions differed significantly across villages (p = 0.00, p = 0.00, p = 0.00, p = 0.23, respectively) (See Additional file 3: Table S3). Removal of stagnant water or covering water bodies was the most frequently reported measure to prevent mosquito breeding (47.9%; 125/261), however, some participants either did not know or mentioned incorrect mosquito breeding prevention measures. A high proportion of participants (98%; 256/261) reported that they adhere to the above-mentioned malaria prevention measures while the rest of the participants (2%, 5/261) either did so sometimes or did not adhere to any measure at all. Of note, 96.9% (253/261) of the study participants reported not to have slept under a bed net the previous night.

### Participants’ reasons for participating in the study

**Fig. 3:**
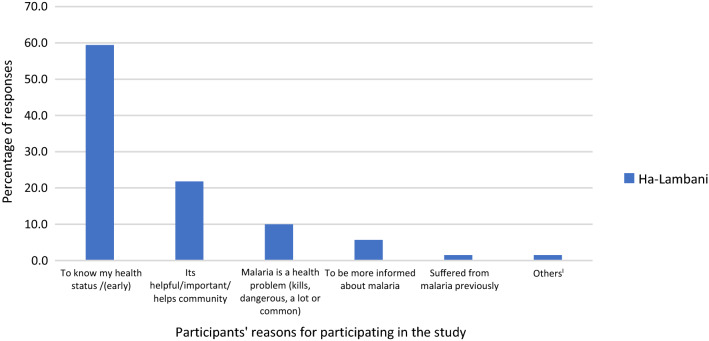
Respondents’ reasons for participating in the study (others included I may have it and not know/others have it and don’t know; no need to travel long distance the clinic to get tested)

The study participants had different reasons for participating in the current study (Fig. 3). A majority of the participants reported the need to know their health status followed by recognizing malaria studies as important or helpful to the community as reasons for participating. Also, the rest of the participants mentioned reasons such as malaria is a problem, they have suffered from malaria or to be more informed about malaria. To a lesser extent, participants mentioned that they will not need to travel long distances to get to the clinic to get tested for malaria.

### Knowledge of malaria transmission, symptoms, treatment and prevention stratified by gender, age, educational level and total household income

Female participants had more knowledge on malaria transmission (77.4%; 151/195) compared to males (27.6%; (44/195) (p = 0.00). Knowledge on malaria transmission knowledge was insignificantly higher for participants with secondary school education than other levels (p = 0.51) and significantly higher in participants aged 30 than in those over 30, the difference being very close to the threshold of statistical significance (p = 0.05). Participants who had resided in the village for 5 years or more had greater knowledge of malaria transmission compared to participants who stayed less than 5 years (p = 0.06).

The study participants’ knowledge on malaria symptoms was significantly higher for females than males (p = 0.00), and significantly lower for participants aged 30 and below compared to those above 30 years (p = 0.02). Moreover, the participants’ knowledge on malaria treatment and prevention was significantly higher for females than males (p = 0.00 and p = 0.00, respectively). Total household income was not associated with participants knowledge on malaria transmission, symptoms, treatment or prevention (see Additional file 4: Table S4).

## Outcome of the focus group discussion

Overall, the focus group discussion concurred with the questionnaires, however, with a few ideas added.

### Knowledge of malaria transmission

The study participants had heard of malaria and correctly associated it with mosquito bites as the mode of transmission. Others mentioned the close proximity of the village to Kruger National Park as a risk factor of malaria. When asked of their general knowledge or view of malaria, many mentioned that poor practice of malaria prevention measures such as uncleanliness, sleeping with open windows at night, poor disposal of empty cans and diapers, leaving small water dams unattended attracts mosquitoes resulting in malaria. Malaria symptoms such as headache, vomiting, and body weakness leading to inability to walk were also mentioned. Some quotes regarding these are briefly described below:

“What I know or think about malaria is that it is caused by mosquitoes. These mosquitoes come after biting animals that we have in our very own Kruger National Park that we live next to. This is how malaria comes about. When the mosquito bites us as humans whether asleep or awake, it vomits the blood from the animals. That blood from the animals has poison, which is now introduced to the human body. That poison affects your body and results in you not feeling well. You may start feeling cold, get a headache or start vomiting. That’s the little that I know.”

“Once a malaria mosquito bites, it can bite a lot of people in that same household.”

“Sleeping with open windows at night makes one to be bitten by mosquitoes carrying the malaria parasite”.

“Also, in this generation, you will find a bucket with diapers in the house, if it doesn’t get full or pop from the bottom or something, it will never be removed or emptied! It will only be emptied once the smell becomes unbearable.”

The participants mentioned the local health clinic, school, family, indoor residual spraying (IRS) team and firsthand experience as the source of malaria information, with more emphasis on the local health clinic. Additionally, in the past, the IRS team would also give medication to those showing malaria symptoms immediately. Participants originally knew malaria as “Mudinyane” which would easily go away once you vomit sometimes, even without taking medication.

### Knowledge of malaria symptoms

The symptoms of malaria were clearly recognized by focus group participants. Additionally, deafness and sleepiness/exhaustion emerged. Although headache was mentioned in the questionairres, it was however further described in the quote below:

“It aches differently from other headaches; you feel it on the forehead and you cannot even walk because of it.’’

“It will ache as pins and needles mainly on the forehead, you will feel like your eyes cannot look up.”

“On your feet, your knees tremble, you cannot walk long distances without resting and feeling like you are losing your breath.”

### Knowledge and practice of malaria prevention measures

Additionally, prevention measures described were using Bunganyunu plant” (*Lippia javanica*) which is kept in the room for a short while before sleeping. The smell is reported to drive away mosquitoes. Lastly, in addition to burning cow dung, burning tissue or egg holder cardboard of which, the smoke drives away the mosquitoes was also mentioned. Regarding bed net usage, only one participant owned a bed net and used it while the rest of the participants did not use bed nets due to the reasons expressed in the quote below:

“We would like to use the bed nets but we don’t have them. We cannot afford it, we can’t use one for everyone in the house, it’s expensive to buy for everyone in the house.” If the government can consider us and give us, we would gladly use it.”

Most of the study participants correctly reported removal of stagnant water or removal of used cow dung (commonly used for soil-based floor decorations), proper disposal of empty cans and diapers or keeping their compound clean as ways to prevent mosquito breeding with removal of stagnant water or covering water bodies with soil as the most frequently reported measure to prevent mosquito breeding. This is supported by the quote below:

“Getting rid of cow dung around the compound, once we do that the mosquito will be ashamed to even come to a clean place.”

“If there are small water dams around the house, I need to fill them with soil so that water is not stagnant.”

In addition, adding bleach (Jik) or salt grains in water drums to keep away mosquitoes emerged as methods to prevent mosquito breeding which were not mentioned from the individual questionnaires.

“Another is to use bleach in rain water we collected into water drums, to keep that water clean.”

“If it rained for example, and I collect water into water drums, I add a granule of salt into the water so that mosquitoes won’t enter and breed.”

### Knowledge of malaria treatment and treatment-seeking behaviour

Focus group participants knew that effective treatments for malaria are available. They further described malaria medication in the following quotations:

“Medication for malaria is a malaria drip (intravenous) containing a colorless water like liquid, pills (which are yellow in color of which the dosage is in hours) and malaria injections”.

“There’s another medication, I just do not know what it is called, it is a drip. They inject you; it is effective. They absorb it from a small bottle using an injection and inject it into the drip. You will not even take an hour before you become conscious again. It is very effective.’’

“The yellow pills are bitter, and they (health workers) recommend that we take them with milk, so they can work faster.”

These yellow pills are actually artemether-lumefantrine. In addition, participants mentioned that in the past, malaria medication would be distributed as pills (white in colour) in the community by malaria IRS workers so that they can use them once malaria symptoms show. These are actually chloroquine or sulfadoxine-pyrimethamine (SP) used in the 1990s for malaria treatment in South Africa) [18]. However, this was later discontinued because girls started using them to terminate pregnancies.

The participants had a positive treatment-seeking behaviour by stating that they would take their child to the clinic if they had symptoms such as fever. This was supported by the quote below:

“I would take the child to the clinic to be tested for malaria.’’

## Further comments by participants

Study participants had further comments regarding malaria summarized as follows:

There should be more awareness programmes on malaria in this area, especially in more malarious villages.

The government/ department of health should provide us with a mobile clinic to serve our villages (Tshihothi, Tshamulavhu and Masetoni) so that we do not have to travel such long distances to get assistance.

They should provide us with bed nets.

They should do house to house malaria testing at least once a month.

They should provide us with our own ambulance.

## Discussion

Results from surveys on knowledge, attitudes and practices are significant in designing, reformulating or improving malaria control programmes, and in identifying markers that demonstrate the effectiveness of a programme [19]. This study assessed community knowledge, attitudes and practices surrounding malaria transmission, symptoms, prevention measures and treatment in Ha-Lambani area of Vhembe District, Limpopo Province, South Africa.

The results of this study showed that the Ha-Lambani community demonstrated adequate knowledge about malaria, transmission, and preventative measures as well as positive treatment-seeking behaviour as observed in other reports from different parts of the world (South East Iran, Tanzania, North Eastern Ethiopia, Mpumalanga Province in South Africa) [17, 20–23]. All the study participants had heard of malaria, this concurs with other studies done in Tanzania, South Africa, Ethiopia, Swaziland, which accounts for 93% to 100%, respectively [14, 20, 23, 24]. The most identified source of information on malaria by the participants was the local health clinic (85%). A similar finding was observed in KwaZulu Natal Province (South Africa), Iran, Zambia, Ethiopia and Swaziland, where 49.8% to 95.5% of the participants reported health organizations as the main source of malaria information [14, 21–23, 25]. However, a smaller proportion (29.1% and 9.6%) accounted for health centres as the source of malaria information in Mpumalanga Province (South Africa) and Tanzania, respectively [19, 23].

The majority (95%) of respondents associated mosquito bites with malaria transmission, which is a common observation in malaria endemic areas. A similar observation ranging between 77 and 99% was reported in KwaZulu Natal Province (South Africa), Ethiopia, Tanzania, Nigeria and Swaziland [14, 17, 20, 25–28]. These are encouraging results in comparison to 33.2% observed in Ethiopia [29]. Amongst other villages, Tshihothi village had more participants that had no knowledge on malaria transmission; more awareness on malaria transmission may be needed in this village. Female participants had more knowledge on malaria transmission (77.4%) compared to males (27.6%) suggesting a need for more education for the males. However, this may be influenced by the small number of male participants in the study. Also, no significant difference on malaria transmission knowledge was observed based on level of education.

The knowledge of malaria symptoms is usually high in areas endemic for malaria where people are aware of the clinical manifestations of the disease [30]^.^ In the current study, the knowledge about malaria symptoms among participants was relatively poor. Although almost all the participants (99%), knew at least one common symptom of malaria as seen in Ethiopia (78%), only 21% correctly identified the 3 most common symptoms of malaria (headache, chills and fever) [1, 26]. In contrast to these findings, over 70% of respondents reported headache, fever or high temperature and chills in Swaziland [25]. The symptoms headache, vomiting and diarrhoea were amongst others the most frequently reported symptoms of malaria. This concurs with a study done by Cox in 2018 which reported headache, diarrhoea, vomiting and shivering or convulsions as the most frequently reported symptoms of malaria [13]. This is contrary to fever and chills reported by Abate in 2013 [20]. Participants also mentioned sweating, loss of appetite, fever and chills; and to a lesser extent fatigue, loss of weight, joint pains and others, while a very small proportion did not know of any symptoms of malaria. This suggests that the participants lack sufficient knowledge of symptoms specific to malaria. This is an important observation as knowledge of signs and symptoms play a particular role in early diagnosis and treatment of disease [31]. More female participants had knowledge on malaria symptoms than males, perhaps male targeted education is needed in the community regarding malaria symptoms. However, this may be influenced by the small number of male participants in the study.

The participants’ knowledge of malaria treatment was extremely low; however, although not the first-line drug for malaria, one participant mentioned quinine as medication for malaria treatment. Majority of the participants were quite aware that effective treatment for malaria is available although they did not know specific names of the anti-malarials. The majority of respondents simply mentioned pills/tablets (describing the colour to be yellow) as medication for the treatment of malaria. This suggests that the community lacks specific details of the medication for malaria treatment that they receive from the local clinic while a small number of the respondents had no knowledge of any form of medication for malaria treatment. Moreover, the participants’ knowledge on malaria treatment was significantly higher for females suggesting more education is needed for the males. However, this may be influenced by the small number of male participants in the study.

Knowledge of malaria prevention measures was high (98%), as observed in Uganda [32]. Wearing long-sleeved clothes was the most prominent malaria prevention measure. This is in contrast with a study done in North Eastern Ethiopia [20]. Historically, African communities have used traditional methods amongst others, in order to keep away mosquitoes. Similarly, in the current study, minority (16.5%) of the study participants reported the use of unproven prevention measures for malaria control such as burning cow dung, or using plants [such as “Musuzungwane” (*Lippia javanica*)]. Congruent with this study, 22% of the study participants as reported by Manana also mentioned these measures [14]. Moreover, almost half of the study participants mentioned getting rid of stagnant water or covering water bodies as measures to prevent mosquito breeding. This concurs with a study done by Deressa et al. in Ethiopia [30]. Similarly, Aderaw and Gedefaw reported that 72.6% of participants mentioned stagnant water as breeding sites for malaria [29].

A high number of the participants reported to adhere to prevention measures; however, only 3% of them reported to have slept under a bed net the previous night. Similarly, Manana et al*.* found in 2017 that only 2% of the study participants in KwaZulu Natal Province (South Africa) used bed nets [14]. In contrast, studies performed in Zimbabwe, Columbia, Ethiopia, Iran and Nigeria found that a higher proportion of the population (17–93%) reported to practice the use of bed nets [20, 33–36]. The poor use of bed nets in the current study was due to lack of bed nets ownership resulting from low economic status by participants as per the focus group discussion [33].

Also, this may be due to the fact that South Africa does not provide bed nets for its citizens as part of the vector control intervention; however, the Department of Health recommends the use of bed nets for personal protection against malaria [14].

The focus group data concurred with the findings from the individual interviews; however, the following additional information was revealed. Participants originally knew malaria as “Mudinyane” which would easily go away once you vomit; sometimes, even without taking medication. The focus group discussion also revealed other unproven malaria prevention measures which are using plants such as “Bunganyunyu plant” in addition to Musudzungwane plant (*Lippia javanica*) of which the smell is reported to drive away mosquitoes. Lastly, in addition to burning cow dung, other unproven prevention measures such as burning tissue or egg holder cardboard which also drives away the mosquitoes by its smoke was also mentioned as seen in a study done by Manana in 2017 [14]. This may be due to perceptions of individual participants based on previous experiences. In this context, further research is necessary in order to determine the effectiveness of these unproven measures regarding malaria prevention as well as motivating community residents to use the conventional measures available [14]. Regarding bed net usage, only one participant owned a bed net and used it while the rest of the focus group participants did not practice its usage because of economic barriers. In addition, adding bleach (Jik) or salt grains in water drums to keep away mosquitoes also emerged as methods to prevent mosquito breeding.

This study could be improved by further investigating whether or not indoor residual spraying in participants’ households have been recently done or not, how often participants get tested for malaria, and how early the participants seek treatment upon onset of symptoms. This study had notable strengths including descriptions of baseline information on malaria perceptions, knowledge, attitudes and practices in the Limpopo Province; as well as adding to malaria awareness in the community by providing informational pamphlets on malaria.

## Conclusion and recommendations

The study not only demonstrates that participants have appropriate knowledge about malaria but also positive treatment-seeking behaviour. However, economic barriers are responsible for the inadequate use of bed nets as an effective prevention measure. Therefore, distribution of bed nets to the community should be considered to improve practice of malaria prevention measures. Also, knowledge of signs and symptoms and appropriate malaria treatment was limited, and initiatives to improve awareness on these topics should be continued. Furthermore, a minority of the community reported the use of unconventional prevention measures. Therefore, further research is necessary (even in other parts of the country endemic for malaria) in order to determine the effectiveness of these unproven prevention measures against malaria as well as motivation to community residents regarding the use of conventional prevention measures available. The implication could also be equivalent in other areas of the country endemic for malaria, thereby indicating a need to consider these aforementioned aspects in national malaria intervention plans with the overall goal to combat or eliminate malaria.

## Supplementary Information

**Additional file 1**: **Table S1**. Details the demographic characteristics of study participants by village.

**Additional file 2**: **Table S2**. Details the knowledge and perception on malaria transmission of study participants.

**Additional file 3**: **Table S3**. Details reported knowledge on malaria prevention and practices of participants by village .

**Additional file 4**: **Table S4**. Details the knowledge of malaria stratified by gender, age, educational level and total household income.

## Data Availability

All data generated or analysed during this study are included in this published article.

## Abbreviations

FGD: Focus Group Discussion
IRS: Indoor Residual Spraying
KAP: Knowledge Attitude and Practices
SP: Sulfadoxine-Pyrimethamine
