# Supplementary material for: Community knowledge, attitudes and practices towards malaria in Ha-Lambani, Limpopo Province, South Africa: a cross-sectional household survey
Source: Malar J. 2021 Apr 17;20:188. doi: 10.1186/s12936-021-03724-z (PMC8052774; doi:10.1186/s12936-021-03724-z)
Supplement: Supplementary file 1 — Additional file 1: Table S1. Details the demographic characteristics of study participants by village. [file 12936_2021_3724_MOESM1_ESM.docx]

Additional file 1 Details the demographic characteristics of study participants by village

| **Village name** | **Masetoni**  **n=86** | **Tshihothi**  **n=41** | **Tshamulavhu**  **n=134** | **Total**  **n=261** |
| --- | --- | --- | --- | --- |
| **Gender** | **n (%)** | **n (%)** | **n (%)** | **n (%)** |
| Male | 18(20.9) | 7(17.1) | 23 (17.2) | 48 (18.4) |
| Female | 68 (79.1) | 34 (82.9) | 111 (82.8) | 213(81.6) |
| **Age (range 18-95)** |  |  |  |  |
| ≤30 | 31(36) | 6 (14.6) | 32 (23.9) | 69 (26.4) |
| 31-50 | 27 (31.4) | 17 (41.5) | 59 (44.) | 103 (39.5) |
| >50 | 28(32.6) | 18 (43.9) | 43 (32.1) | 8) 34.1) |
| Mean |  |  |  | 44.1 |
| Median |  |  |  | 40 |
| Mode |  |  |  | 29 |
| SD |  |  |  | 18.1 |
| **Marital Status** |  |  |  |  |
| Never Married | 86(100) | 12(29.3) | 45(33.6) | 144(55.2) |
| Married | 0 (0) | 27(65.9) | 80(59.7) | 107 (41.0) |
| Divorced | 0 (0) | 1(2.4) | 3(2.2) | 4 (1.5) |
| Widowed | 0 (0) | 1(2.4) | 5(3.7) | 6 (2.3) |
| **Nationality** |  |  |  |  |
| South African | 86(100) | 41(100) | 134(100) | 261(100) |
| **Ethnicity** |  |  |  |  |
| Vha-Venda | 85(98.8) | 41(100) | 134(100) | 261(99.6) |
| Va-Tsonga | 1(1.2) | 0(0) | 0(0) | 1(0.4) |
| **Language** |  |  |  |  |
| Tshivenda | 86 (100) | 41(100) | 134(100) | 261(100) |
| **Religion** |  |  |  |  |
| Christian | 77 (89.5) | 37(90.2) | 129(96.3) | 243(93.1) |
| Traditional | 7(8.1) | 4(9.8) | 3(2.2) | 14(5.4) |
| Other^a^ | 2(2.3) | 0(0) | 2(1.5) | 4(1.5) |
| **Family Position** |  |  |  |  |
| Father | 19(22) | 8(19.5) | 17(12.6) | 44(16.8) |
| Mother | 56(65) | 24(58.5) | 103(76.3) | 183(69.8) |
| Legal guardian | 1(1) | 0(0) | 0(0) | 1(0.4) |
| Young adult | 10(12) | 9(22) | 15(11.1) | 34(13.0) |
| **Level of education** |  |  |  |  |
| No formal education | 20(23) | 9(22) | 25(18.7) | 54(20.7) |
| Primary level | 22(26) | 15(36.6) | 30(22.4) | 67(25.7) |
| Secondary level | 41(48) | 16(39) | 75(56) | 132(50.6) |
| Tertiary level | 3(3) | 1(2.4) | 4(3) | 8(3.1) |
| **Occupation** |  |  |  |  |
| Unemployed | 62(72.1) | 27(65.9) | 98(73.1) | 187(71.6) |
| Employed | 8(9.3) | 2(4.9) | 5(3.7) | 15(5.7) |
| Government grant dependent | 16(18.6) | 11(26.8) | 31(23.1) | 58(22.2) |
| Other^b^ | 0(0) | 1(2.4) | 0(0) | 1(0.40 |
| **Total household income** |  |  |  |  |
| <3000 | 80(93.0) | 40(97.6) | 130(97.0) | 250(95.8) |
| 3000-10000 | 5(5.8) | 1(2.4) | 2(1.5) | 8(3.1) |
| <10000 | 1(1.2) | 0(0) | 2(1.50 | 3(1.1) |
| **Malaria history: Past 3 months** |  |  |  |  |
| Yes | 1(1.2) | 2(4.9) | 2(1.5) | 5(1.9) |
| No | 85(98.8) | 39(95.1) | 132(98.5) | 256(98.1) |
| **Past 5 years** |  |  |  |  |
| Yes | 56(65.1) | 31(75.6) | 58(43.0) | 145(55.6) |
| No | 30(34.9) | 10(24.4) | 77(57.0) | 116(44.4) |
| **Fever History: Past 3 days** |  |  |  |  |
| Yes | 4(4.7) | 1 (2.4) | 5(3.7) | 10(3.8) |
| No | 82(95.3) | 40(97.6) | 129(96.3) | 251(96.2) |

Other^a^ represents neither, traditional or Christian. Other^b^ included government grant dependent and pensioner.
