# Supplementary material for: Community knowledge, attitudes and practices towards malaria in Ha-Lambani, Limpopo Province, South Africa: a cross-sectional household survey
Source: Malar J. 2021 Apr 17;20:188. doi: 10.1186/s12936-021-03724-z (PMC8052774; doi:10.1186/s12936-021-03724-z)
Supplement: Supplementary file 2 — Additional file 2: Table S2. Details the knowledge and perception on malaria transmission of study participants. [file 12936_2021_3724_MOESM2_ESM.docx]

| **Village name** | **Masetoni**  **n= 86** | **Tshihothi n= 41** | **Tshamulavhu**  **n= 134** | **Total n= 261** | | **p value** |
| --- | --- | --- | --- | --- | --- | --- |
| **1.1 Have you heard of malaria?** | **n (%)** | **n (%)** | **n (%)** | | **n (%)** | **(α= 0.05)** |
| Yes | 86(100) | 41(100) | 134(100) | | 261(100) | - |
| No | 0(0) | 0(0) | 0(0) | | 0(0) | **-** |
| **1.2. Knowledge of malaria transmission** |  |  |  | |  |  |
| Mosquito bites | 80(93) | 36(87.8) | 131(97.8) | | 247(94.6) | **P=0.03** |
| Don’t know | 5(5.8) | 5(12.2) | 2(1.5) | | 12(4.6) | **P=0.01** |
| Other transmission^c^ | 1(1.2) | 0(0) | 1(0 | | 2(0.80 | - |
| **1.3. What is your general view of malaria?** |  |  |  | |  |  |
| It kills, dangerous, a lot or problematic | 80(93) | 39(95.1) | 124(92.5) | | 243(93.1) | P=0.84 |
| Mentions malaria symptom(s) | 1(1.2) | 0(0) | 4(3) | | 5(1.9) | - |
| Mentions prevention method(s) | 2(2.3) | 1(2.4) | 5(3.7) | | 8(3.1) | P=0.82 |
| No comment | 3(3.5) | 1(2.4) | 1(0.7) | | 5(1.9) | P=0.34 |
| **1.4. What can you say about its occurrence in the Ha-Lambani area in years** |  |  |  | |  |  |
| It kills, dangerous, a lot or problematic | 75(87.2) | 40(97.6) | 131(97.8) | | 246(94.30 | **P=0.00** |
| Mentions malaria symptom(s) | 3(3.5) | 0(0) | 0(0) | | 3(1.1) | -- |
| Mentions prevention method(s) | 7(8.1) | 0(0) | 3(2.2) | | 10(3.8) | -- |
| Others^d^ | 1(1.2) | 1(2.4) | 0(0) | | 2(0.80 | -- |
| **1.5. Length of stay in Ha-Lambani area in years** |  |  |  | |  |  |
| ≤4 | 3(3.5) | 1(2.4) | 4(3) | | 8(3.1) | P=0.95 |
| ≥5 | 83(96.5) | 40(97.6) | 130(97.) | | 253(96.9) | P=0.95 |
| **1.6 Relating malaria to Kruger National Park** |  |  |  | |  |  |
| Yes | 3(3.5) | 0(0) | 2(1.5) | | 5(1.9) | - |
| No | 83(96.50 | 41(100) | 132(98.5) | | 256(98.1) | - |
| **Total** | **86(100)** | **41(100)** | **134(100)** | | **261** |  |

Additional file 2 Details the knowledge and perception on malaria transmission of study participants

Other**^c^** transmission routes included drinking bad water and other**^d^** perceptions of malaria in the Ha-Lambani village specifically included refusing to use the same alcohol cup as a person with malaria and no comment. Chi-square test for differences in prevalence across villages.

**TABLE 2:** Reported knowledge and perception on malaria transmission; and its occurrence in Ha-Lambani area by village.
