# Supplementary material for: Community knowledge, attitudes and practices towards malaria in Ha-Lambani, Limpopo Province, South Africa: a cross-sectional household survey
Source: Malar J. 2021 Apr 17;20:188. doi: 10.1186/s12936-021-03724-z (PMC8052774; doi:10.1186/s12936-021-03724-z)
Supplement: Supplementary file 3 — Additional file 3: Table S3. Details reported knowledge on malaria prevention and practices of participants by village . [file 12936_2021_3724_MOESM3_ESM.docx]

| **Village name** | **Masetoni n=86** | **Tshihothi n=41** | **Tshamulavhu n=134** | **Total n=261** | **p value** |
| --- | --- | --- | --- | --- | --- |
| **Characteristic** | **n (%)** | **n (%)** | **n (%)** | **n (%)** | **α= 0.05** |
| **2.3. Knowledge of malaria prevention** |  |  |  |  |  |
| Using bednets | 24(27.9) | 3(7.3) | 35(26.1) | 62(23.8) | **P=0.03** |
| Burning cow dung/ Musuzungwane plant leaves (*Lippia javanica*) | 10(11.6) | 5(12.2) | 28(20.9) | 43(16.5) | P=0.14 |
| Removal of dirty or stagnant water | 13(15.1) | 0(0) | 9(6.7) | 22(8.4) |  |
| Removal of used cow dung | 0(0) | 0(0) | (2.2)3 | 3(1.1) |  |
| Wearing long sleeved clothes | 42(48.8) | 1(2.4) | 59(44) | 102(39.1) | **P=0.00** |
| using mosquito repellants | 5(5.8) | 3(7.3) | 5(3.7) | 13(5) | P=0.59 |
| Using mosquito coils | 5(5.8) | 1(2.4) | 12(9) | 18(6.9) | P=0.32 |
| Burying empty cans | 7(8.1) | 1(2.4) | 5(3.7) | 13(5) | P=0.24 |
| Keeping cleanliness | 10(11.6) | 1(2.4) | 13(9.7) | 24(9.2) | P=0.23 |
| Allow IRS workers to spray your house | 8(9.3) | 3(7.3) | 8(6) | 19(7.3) | P=0.27 |
| Closing windows | 8(9.3) | 4(9.8) | 4(3) | 16(6.1) | P=0.09 |
| Clinic/ treatment | 1(1.2) | 2(4.9) | 3(2.2) | 6(2.3) | P=0.42 |
| Don’t know | 0(0) | 3(7.3) | 1(0.7) | 4(1.5) | -- |
| Other^h^ | 1(1.2) | 1(2.4) | 6(4.5) | 8(3.1) | P=0.37 |
| **3.1. Knowledge of preventing mosquito breeding** |  |  |  |  |  |
| Removal of dirty, stagnant water or covering water holes | 47(54.7) | 1(2.4) | 77(57.5) | 125(47.9) | **P=0.00** |
| Removal of used cow dung | 10(11.6) | 27(65.9) | 25(18.7) | 62(23.8) | **P=0.00** |
| Proper tin disposal | 29(33.7) | 1(2.4) | 20(14.9) | 50(19.2) | **P=0.00** |
| Clean compound | 8(9.3) | 8(19.5) | 25(18.7) | 33(12.6) | P=0.14 |
| Don’t know | 3(3.5) | 1(2.4) | 5(3.7) | 9(3.4) | P=0.92 |
| Incorrect measures^i^ | 14(16.3) | 10(24.4) | 5(3.7) | 29(11.1) | **P=0.00** |
| **3.2. Adherence to preventative measures** |  |  |  |  |  |
| Yes | 85(98.8) | 41(100) | 130(97) | 256(98) | - |
| No | 0(0) | 0 | 1(0.7) | 1(0) | - |
| Sometimes | 1(1.2) | 0 | 3(2.2) | 4(2) | **-** |
| - 1. **Did you sleep under a bednet last night?** |  |  |  |  |  |
| Yes | 2(2.3) | 0 | 6(4.5) | 8(3.1) | - |
| No | 84(97.7) | 41(100) | 128(95.5) | 253(96.9) | - |

Additional file 3 Details reported knowledge on malaria prevention and practices of participants by village

Percentage total exceed 100 because of multiple responses. Other^h^ prevention measures included the use of clean water, fans, clean food, drinking stoney soft drink to prevent malaria. Incorrect prevention measures^i^ included the use of bednets, closing windows, shaking off curtains, wearing long sleeved clothes, burning mosquito coils or using mosquito repellants. Chi-square test for differences in prevalence across villages.

| **Village name** | **Masetoni n=86** | **Tshihothi n=41** | **Tshamulavhu n=134** | **Total n=261** | **p value** |
| --- | --- | --- | --- | --- | --- |
| **Characteristic** | **n (%)** | **n (%)** | **n (%)** | **n (%)** | **α= 0.05** |
| **2.3. Knowledge of malaria prevention** |  |  |  |  |  |
| Using bednets | 24(27.9) | 3(7.3) | 35(26.1) | 62(23.8) | **P=0.03** |
| Burning cow dung/ Musuzungwane plant leaves (*Lippia javanica*) | 10(11.6) | 5(12.2) | 28(20.9) | 43(16.5) | P=0.14 |
| Removal of dirty or stagnant water | 13(15.1) | 0(0) | 9(6.7) | 22(8.4) |  |
| Removal of used cow dung | 0(0) | 0(0) | (2.2)3 | 3(1.1) |  |
| Wearing long sleeved clothes | 42(48.8) | 1(2.4) | 59(44) | 102(39.1) | **P=0.00** |
| using mosquito repellants | 5(5.8) | 3(7.3) | 5(3.7) | 13(5) | P=0.59 |
| Using mosquito coils | 5(5.8) | 1(2.4) | 12(9) | 18(6.9) | P=0.32 |
| Burying empty cans | 7(8.1) | 1(2.4) | 5(3.7) | 13(5) | P=0.24 |
| Keeping cleanliness | 10(11.6) | 1(2.4) | 13(9.7) | 24(9.2) | P=0.23 |
| Allow IRS workers to spray your house | 8(9.3) | 3(7.3) | 8(6) | 19(7.3) | P=0.27 |
| Closing windows | 8(9.3) | 4(9.8) | 4(3) | 16(6.1) | P=0.09 |
| Clinic/ treatment | 1(1.2) | 2(4.9) | 3(2.2) | 6(2.3) | P=0.42 |
| Don’t know | 0(0) | 3(7.3) | 1(0.7) | 4(1.5) | -- |
| Other^h^ | 1(1.2) | 1(2.4) | 6(4.5) | 8(3.1) | P=0.37 |
| **3.1. Knowledge of preventing mosquito breeding** |  |  |  |  |  |
| Removal of dirty, stagnant water or covering water holes | 47(54.7) | 1(2.4) | 77(57.5) | 125(47.9) | **P=0.00** |
| Removal of used cow dung | 10(11.6) | 27(65.9) | 25(18.7) | 62(23.8) | **P=0.00** |
| Proper tin disposal | 29(33.7) | 1(2.4) | 20(14.9) | 50(19.2) | **P=0.00** |
| Clean compound | 8(9.3) | 8(19.5) | 25(18.7) | 33(12.6) | P=0.14 |
| Don’t know | 3(3.5) | 1(2.4) | 5(3.7) | 9(3.4) | P=0.92 |
| Incorrect measures^i^ | 14(16.3) | 10(24.4) | 5(3.7) | 29(11.1) | **P=0.00** |
| **3.2. Adherence to preventative measures** |  |  |  |  |  |
| Yes | 85(98.8) | 41(100) | 130(97) | 256(98) | - |
| No | 0(0) | 0 | 1(0.7) | 1(0) | - |
| Sometimes | 1(1.2) | 0 | 3(2.2) | 4(2) | **-** |
| - 1. **Did you sleep under a bednet last night?** |  |  |  |  |  |
| Yes | 2(2.3) | 0 | 6(4.5) | 8(3.1) | - |
| No | 84(97.7) | 41(100) | 128(95.5) | 253(96.9) | - |
