# Supplementary material for: Community knowledge, attitudes and practices towards malaria in Ha-Lambani, Limpopo Province, South Africa: a cross-sectional household survey
Source: Malar J. 2021 Apr 17;20:188. doi: 10.1186/s12936-021-03724-z (PMC8052774; doi:10.1186/s12936-021-03724-z)
Supplement: Supplementary file 4 — Additional file 4: Table S4. Details the knowledge of malaria stratified by gender, age, educational level and total household income. [file 12936_2021_3724_MOESM4_ESM.docx]

| **Characteristic** | | **Total n=261** | **Knowledge on malaria** | **P value** | **Knowledge on malaria transmission** | **P value** | **Knowledge on malaria Symptoms** | **P value** | **Knowledge on malaria treatment** | **P value** | **Knowledge on malaria prevention** | **P value** |
| --- | --- | --- | --- | --- | --- | --- | --- | --- | --- | --- | --- | --- |
| **Gender** | Male | 48(18.39) | 48(18.39) | P=0. 76 | 44(22.6) | P=0.00 | 94(36.6) | P=0.00 | 73(28.40 | **P=0.00** | 74(28.8) | **P=0.00** |
|  | Female | 213(81.61) | 213(81.61) | P=0. 76 | 151(77.4) | P=0.00 | 163(63.4) | P=0.00 | 184(71.6) | **P=0.00** | 183(71.2) | **P=0.00** |
| **Total** |  | **261** | **261** |  | **195** |  | **257** |  | **257** |  | **257** |  |
| **Age** | ≤30 | 69(26.4) | 69(26.44) | P=0.02 | 66(26.5) | P=0.05 | 75(29.2) | P=0.02 | 67(26.1) | P=0.166 | 68(26.5) | P=0.31 |
|  | 31-50 | 103(39.5) | 103(39.46) | P=0.38 | 100(40.2) | P=0.12 | 95(36.96) | P=0.12 | 98(38.1) | P=0.10 | 102(39.7) | P=0.16 |
|  | >50 | 89(34.1) | 89(34.1) | P=0.35 | 83(33.3) | P=0.73 | 87(33.85) | P=0.53 | 92(35.8) | P=0.33 | 87(33.9) | P=0.73 |
| **Total** |  | **261** | **261** |  | **249** |  | **257** |  | **257** |  | **257** |  |
| **Educational level** | No formal education | 54(20.7) | 54(20.7) | P=0.71 | 47(18.9) | P=0.37 | 54(21.7) | P=0.06 | 53(20.62) | P=0.54 | 52(20.3) | p=0.61 |
|  | Primary | 67(25.7) | 67(25.7) | P=0.11 | 67(26.9) | P=0.13 | 59(23.7) | P=0.13 | 66(25.68) | P=0.29 | 64(25) | p=0.17 |
|  | secondary | 132(50.6) | 132(50.6) | P=0.00 | 116(46.6) | P=0.51 | 125(50.2) | P=0.09 | 132(51.36) | P=0.79 | 130(50.8) | P=0.18080 |
|  | Tertiary | 8(3.1) | 8(3.10 | P=0.95 | 19(7.6) | P=0.06 | 11(4.4) | P=0.19 | 6(2.33) | P=1.109 | 10(3.91) | P=0.85 |
| **Total** |  | **261** | **261** |  | **249** |  | **249** |  | **257** |  | **256** |  |
| **Total household income** | <3000 | 250(95.8) | 250(95.8) | P=0.29 | 239(94.1) | P=0.07 | 244(94.9) | P=0.21 | 246(95.7) | P=0.83 | 248(96.5) | - |
|  | 3000-10000 | 8(3.1) | 8(3.1) | P=0.19 | 12(4.7) | P=0.03 | 8(3.1) | P=0.16 | 7(2.7) | P=1.10 | **6** | - |
|  | >10000 | 3(1.1) | 3(1.1) | - | 3(1.2) | - | 5(1.9) | - | 4(1.6) | - | 3(2.3) | - |
| **Total** |  | **261** | **261** |  | **254** |  | **257** |  | **257** |  | **257** |  |
| **Length of stay in Ha-Lambani area in years** | <4 | 8(3.07) | 8(3.07) | P=0.60 | 8(4.52) | p=0.00 | 9(3.46) | **P=0.92** | 9(3.5) | **P=0.75** | 13(5) | **P=0.30** |
|  | ≥5 | 253(96.93) | 253(96.93) | P=0.60 | 169(95.5) | P=0.00 | 251(96.54) | **P=0.92** | 251(97.67) | **P=0.75** | 247(95) | **P=0.30** |
|  |  | **261** | **261** |  | **177** |  | **260** |  | **260** |  | **260** |  |

Additional file 4 Details the knowledge of malaria stratified by gender, age, educational level and total household income
